# Hyaluronic Acid Hybrid Cooperative Complexes Nearing 10 Years of Use: Update on Safety Assessment Based on Post‐Marketing Surveillance Data

**DOI:** 10.1111/jocd.70197

**Published:** 2025-07-14

**Authors:** Giovanni Salti, Antonello Tateo, Daniel Cassuto, Francesca Arrigoni, Chiara Godina, Federica Mazzola, Clara Cigni, Franco Grimolizzi, Gilberto Bellia

**Affiliations:** ^1^ Medlight Institute Florence Italy; ^2^ Private Practice Milan and Pavia Italy; ^3^ Private Practice Israel; ^4^ Private Practice Milan Italy; ^5^ Agorà Clinical Educational Centre Milan Italy; ^6^ IBSA Institut Biochimique SA Lugano Switzerland; ^7^ IBSA Farmaceutici Italia Srl Lodi Italy

**Keywords:** hyaluronic acid, hybrid cooperative complexes, injectable complications, post‐marketing safety

## Abstract

**Background:**

Hybrid cooperative complex hyaluronic acid (HCC HA) is a novel, widely used injectable developed using the patented NAHYCO Hybrid Technology for the treatment of the face and body, respectively.

**Aim:**

To examine the safety profile of HCC HA from global post‐marketing surveillance data.

**Methods:**

Post‐marketing adverse events (AEs) reported for HCC HA injected to the face (Profhilo, HCC‐HA_PROF_, IBSA Farmaceutici Italia Srl, Italy) from January 1, 2018 to October 31, 2023 and to the body (Profhilo Body, HCC‐HA_PROF‐B_, IBSA Farmaceutici Italia Srl, Italy) from January 1, 2020 to October 31, 2023 were analyzed. Patient exposure and the proportion of exposed patients with a safety complaint were also estimated.

**Results:**

The total number of patients exposed to HCC‐HA_PROF_ and HCC‐HA_PROF‐B_ globally was projected to be 1 091 956 and 27 692, respectively. There was a total of 371 AEs recorded for HCC‐HA_PROF_, and 11 AEs reported for HCC‐HA_PROF‐B_, corresponding to 0.034% and 0.040% of the patients, respectively. The most common AEs were edema, erythema, and discomfort, which are consistent with the manner and location of administration, and the AEs are frequently reported following treatment. The proportion of exposed patients with a safety complaint was low, with 0.026%–0.050% and 0%–0.013% of patients exposed to HCC‐HA_PROF_ or HCC‐HA_PROF‐B_ experiencing a safety complaint, respectively.

**Conclusions:**

Post‐marketing surveillance indicated favorable safety profiles for HCC‐HA_PROF_ and HCC‐HA_PROF‐_, with the most common AEs expected and/or related to the method or site of administration.

## 1 Introduction

Skin aging is a complex and continuous process that affects skin function and appearance [1, 2], with onset and progression varying between individuals [3]. Intrinsic aging of the skin occurs as a natural consequence of physiological changes determined by genetics. Extrinsic factors are numerous, including exposure to sunlight, pollution, smoking, and rapid changes in body weight (e.g., diet, pregnancy) [4, 5]. Skin aging can lead to skin roughness and laxity, in both the face and other body sites, including arms, knees, abdomen, neck, décolleté, and hands [4, 5, 6].

Hyaluronic acid (HA)‐based injectable treatments are widely used in aesthetic medicine for correcting wrinkles, skin laxity, and roughness in several body areas, including the face, inner arm, abdomen, and knees [4, 7, 8, 9, 10]. HA is found throughout various human tissues, such as the skin, eyes, connective tissue, and synovium [11]. Due to its anionic properties, HA attracts water to provide volume and structural support [7, 12]. Aging contributes to decreased production of HA and collagen in the skin, resulting in decreased elasticity and skin aging [11]. HA products work to counteract aging by replacing lost volume, improving skin elasticity and hydration, and have also been shown to increase collagen production [7]. HA has other properties that make it an attractive agent for aesthetic medicine; it is biocompatible, non‐immunogenic, and biodegradable via hyaluronidase [13, 14].

Several HA formulations are available, differing in degree of cross‐linking, gel consistency properties, and concentration [14], and have shown efficacy in several body areas, including the face, inner arm, abdomen and knees [4, 7, 8, 9, 10]. HCC‐HA_PROF_ (Profhilo, IBSA Farmaceutici Italia srl, Italy) received Conformité Européenne (CE) marking in 2015 and is a novel HA preparation based on stable hybrid cooperative complexes (HCC‐HA), which is the first product developed by NAHYCO Hybrid Technology, an innovative patent protected thermal production process. Thanks to NAHYCO Technology, HCC‐HA_PROF_ can deliver a higher concentration of HA (32 mg of low molecular weight HA + 32 mg of high molecular weight HA into 2 mL) compared to traditional cross‐linked fillers. Moreover, HCC‐HA shows peculiar characteristics in terms of rheology; their high flowability (tanδ > 1) also allows HCC‐HA to optimally integrate once injected into the tissues better than synthetic injectables. Moreover, HCC‐HA_PROF_ also shows an optimal cohesivity value equal to 4.5 assessed by the Gavard‐Sundaram scale [15] (scale ranging from 1 = low cohesivity; 5 = high cohesivity), ensuring natural‐looking results. In this way, HCC‐HA exerts their bioremodeling action, defined as the process that reverses tissue laxity, promoting the homeostasis and vitality of the extracellular matrix components, essential for the function of several cell types, including keratinocytes, fibroblasts, adipocytes and myocytes [16]. Based on HCC‐HA, HCC‐HA_PROF_ is indicated for the treatment of the face and for the treatment of the malar‐zygomatic and submalar areas. HCC‐HA_PROF‐B_ (Profhilo Body, IBSA Farmaceutici Italia srl, Italy) is also an HCC preparation produced using the same technology and contains 48 mg of low molecular weight HA + 48 mg of high molecular weight HA in 3 mL. In clinical studies, HCC‐HA_PROF_ improved wrinkle appearance and reversed skin laxity. HCC‐HA_PROF‐B_ improved skin elasticity and skin roughness for the inner arm, abdomen, knees, and hands of participants with mild to moderate skin roughness and laxity. Importantly, both treatments exhibited positive safety and tolerability profiles together with long‐lasting results [4, 17].

Evidence indicates that the most common adverse events (AEs) associated with HA injectable treatments are related to the injection procedure and are usually transient. However, rare AEs may occur which can lead to severe complications, such as vascular occlusion [18, 19]. Given the importance of ongoing safety monitoring, the objective of this analysis was to examine the safety profile of HCC‐HA_PROF_ and HCC‐HA_PROF‐B_ derived from worldwide post‐marketing surveillance data.

## 2 Materials and Methods

### 2.1 Post‐Marketing Data Collection

HCC‐HA_PROF_ is a 2 mL prefilled syringe containing 32 mg of high‐molecular weight HA and 32 mg of low‐molecular weight HA. HCC‐HA_PROF‐B_ is a 3 mL prefilled syringe containing 48 mg of high‐molecular weight HA and 48 mg of low‐molecular weight HA (IBSA Farmaceutici Italia srl, Italy). Both HCC‐HA_PROF_ and HCC‐HA_PROF‐B_ injection techniques require the use of the BioAesthetic Point (BAP) technique, as per product IFU. The BAP technique requires the intradermic injection of the product using a 29G × ½″ (0.33 × 12 mm) needle. Five injection points (0.2 mL of the product/each bolus) for each side of the face in the malar and submalar regions have been identified, while 10 injection points (0.2 mL of the product/each bolus) have been identified for the treatment with HCC‐HA_PROF_ of the face and neck, respectively. HCC‐HA_PROF‐B_ requires intradermal injection with a dosage of 0.3 mL for each bolus. The BAP approach is used to determine 10 injection locations that correspond to the anatomical region being targeted.

All spontaneous post‐marketing AE reports were sent to the IBSA Farmaceutici Italia global safety database from physicians, healthcare professionals and consumers worldwide who treated patients with HCC‐HA_PROF_ (between January 1, 2018 to October 31, 2023) or HCC‐HA_PROF‐B_ (January 1, 2020 [the year the product was first commercialized] to October 31, 2023) and were subsequently analyzed. In the analyses, safety results were reported by specific periods for HCC‐HA_PROF_ (January 1, 2018 to October 31, 2023) and HCC‐HA_PROF‐B_ (January 1, 2020 to October 31, 2023).

### 2.2 Statistical Analysis

The number of sold syringes was obtained from sales data and used to estimate patient exposure. The recommended dosing regimen for both HCC‐HA_PROF_ and HCC‐HA_PROF‐B_ is an initial cycle of two treatment sessions at 30‐day intervals, followed by maintenance treatment every 2 months if necessary. Patient exposure was estimated by assuming that the highest number of syringes that could be used by a patient for a year‐long cycle of treatment was seven, that is, two during the first 2 months and then one every 2 months. Accordingly, the number of patients exposed = number of syringes sold/7 [17]. The complaint rate for exposed patients was also estimated and expressed as a percentage, that is, ([number of AEs/number of exposed patients] × 100).

## 3 Results

### 3.1 HCC‐HA_PROF_ Safety Results

The number of AEs per main category for HCC‐HA_PROF_ is presented in Table 1. Between January 1, 2018 to October 31, 2023, a total of 371 AEs were reported. AEs for the categories of “injection site conditions” (*n* = 201) and “skin conditions” (*n* = 81) were the most common type of AE reported (Table 1). The most commonly reported AEs (those which had ≥ 5 events) over the time period were “administration site conditions” (face oedema, *n* = 8; injection site erythema, *n* = 8; injection site nodule, *n* = 5; injection site oedema, *n* = 5; injection site pain, *n* = 10; injection site swelling, *n* = 21; nodule, *n* = 6; oedema, *n* = 12; pain, *n* = 7; swelling, *n* = 22; swelling face, *n* = 24), skin conditions (dry skin, *n* = 5; erythema, *n* = 21; pruritus, *n* = 8; rash, *n* = 5; skin wrinkling, *n* = 5), eye swelling or oedema (periorbital swelling, *n* = 5), and procedural complications (off‐label use, *n* = 5). Events such as swelling, redness (erythema) and pain are typical AEs expected after treatment and are listed in the HCC‐HA_PROF_ product information leaflet. These AEs are considered normal if they disappear within a short period of time and may be linked to injection technique and the water retaining properties of HA. The incidence of vascular AEs such as embolism and vascular occlusion was low (*n* = 1 for both events).

**TABLE 1:** Total adverse events and estimated proportion of exposed patients with an adverse event for HCC‐HA_PROF_ by AEs main categories, January 1, 2018 to October 31, 2023.

AEs main categories	Specific term	No. of total events	Estimated proportion of exposed patients who experienced an AE^b^
Administration site conditions	Injection site pain	10	0.0009
	Injection site swelling	21	0.0019
	Oedema	12	0.0011
	Swelling	22	0.0020
	Swelling face	24	0.0022
	Face oedema	8	0.0007
	Injection site erythema	8	0.0007
	Injection site nodule	5	0.0005
	Injection site oedema	5	0.0005
	Nodule	6	0.0005
	Pain	7	0.0006
	Others^a^	64	0.0059
	Subtotal	192	0.0176
Skin conditions	Dry skin	5	0.0005
	Erythema	21	0.0019
	Pruritus	8	0.0007
	Rash	5	0.0005
	Skin wrinkling	5	0.0005
	Others^a^	35	0.0032
	Subtotal	79	0.0072
Eye swelling/oedema	Periorbital swelling	5	0.0005
	Others^a^	14	0.0013
	Subtotal	19	0.0017
Procedural complications	Off‐label use	5	0.0005
	Others^a^	14	0.0013
	Subtotal	19	0.0017
General discomfort	Burning/dizziness/headache	8	0.0007
	Others^a^	5	0.0005
	Subtotal	13	0.0012
Sum of conditions with < 10 AEs reported		39	0.0036

Abbreviation: AE, adverse event.

^a^ Others include conditions with < 5 AEs reported.

^b^ Proportion of exposed patients experiencing an AE was estimated and expressed as a percentage that is, ([number of AEs/number of exposed patients] × 100).

There was a total of 11 quality complaints notified together with AEs for HCC‐HA_PROF_ over the specified period, which included ineffective device (*n* = 4), issue with product quality (*n* = 2), device malfunction (*n* = 1), physical contamination (*n* = 1), product deposits (*n* = 1), leakage (*n* = 1) and abnormal odor (*n* = 1).

**FIGURE 1:**
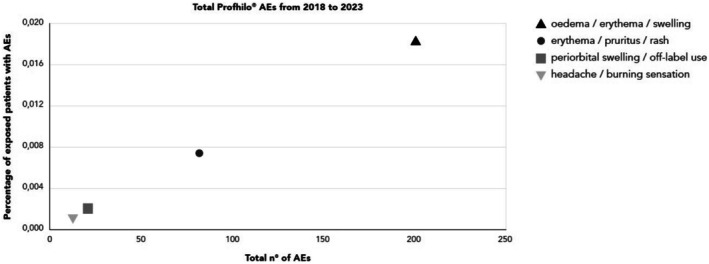
Total adverse events for HCC‐HA_PROF_ versus percentage of exposed patients with AEs by AEs main categories, January 1, 2018 to October 31, 2023. AEs with a ≥ 10 total events have been reported and grouped as indicated in the figure legend: Oedema, erythema and swelling AEs (201 total events); Skin erythema, pruritus, rash (81 total events); Periorbital swelling or procedural complication/off‐label use (19 total events/each); Headache and burning sensation (13 total events).

Based on worldwide sales data, the number of syringes sold for HCC‐HA_PROF_ increased substantially from 2018 to 2023, with year‐on‐year increases observed (Table 1). The recommended treatment for both HCC‐HA_PROF_, as well as HCC‐HA_PROF‐B_, requires two injections 30 days apart and a follow‐up visit 2 months after the last injection to evaluate maintenance sessions. A recent clinical study [17] has demonstrated the long‐term safety of the treatment after 7 injections performed in 1 year. Based on the results published by Sparavigna et al. [17] and comparably to the analysis performed by previous pharmacovigilance reports [18], a maximum number of 7 syringes used for each patient has been considered to perform the statistical analysis for the present post‐marketing assessment. Therefore, the following calculation has been used to obtain the proportion of exposed patients with a safety complaint in 1 year: number of patients exposed = number of syringes sold/7. Therefore, the number of patients exposed to treatment followed a similar trend to the sales data, with global cumulative patient exposure across the specified time periods estimated at 1 091 956. Given that most patients often do not need seven injections in a single year and hence get fewer injections, it is likely that this value has been underestimated. The estimated proportion of exposed patients who experienced AEs between 2018 and 2023 was low for all the categories of AEs analyzed, even though the number of exposed patients is likely underestimated compared to the real number and the maximum number of syringes for each patient has been taken into consideration (Table 1). More specifically, when the principal cause of AEs was analyzed and compared with the percentage of exposed patients, results demonstrated that the estimated proportion of patients with AEs is extremely low (< 0.020%) compared to the absolute number of total AEs reported (Figure 1). Although a total number of 201 cases of face edema, erythema, and swelling (injection site conditions), 81 cases for skin erythema, pruritus, and rash (skin conditions), 19 cases for periorbital swelling, 19 cases for off‐label use, and 13 cases for headache and burning sensations were reported, they only count for 0.018%, 0.007%, 0.002%, and 0.001% of total exposed patients, respectively. Other AEs categories, which count for less than 10 total events in the period comprise between January 2018 and October 2023, only represent 0.003% of the total exposed patients.

The proportion of exposed patients with a safety complaint was also extremely low, with 0.026%–0.050% of patients experiencing a safety complaint across the time periods assessed. The number of patients with a safety complaint generally increased with exposure, but the complaint rate (%) remained low (Table 2).

**TABLE 2:** Sales data, patient exposure, and complaint rate for HCC‐HA_PROF_ between January 1, 2018 and October 31, 2023.

Year	HCC‐HA_PROF_			
	No. of syringes sold	Estimated no. of patients exposed^a^	No. of safety complaints	Estimated proportion of exposed patients with a safety complaint (%)
2018	300 989	42 998	13	0.030
2019	714 973	102 139	30	0.029
2020	809 223	115 603	37	0.032
2021	1 423 433	203 348	63	0.031
2022	2 179 514	311 359	82	0.026
2023	2 215 560	316 509	158	0.050

^a^ Patient exposure was estimated by assuming that the highest number of syringes that could be used by a patient for a year‐long cycle of treatment was seven, as demonstrated by Sparavigna et al. Therefore, the number of patients exposed = number of syringes sold/7.

### 3.2 HCC‐HA_PROF_ _‐B_ Safety Results

**FIGURE 2:**
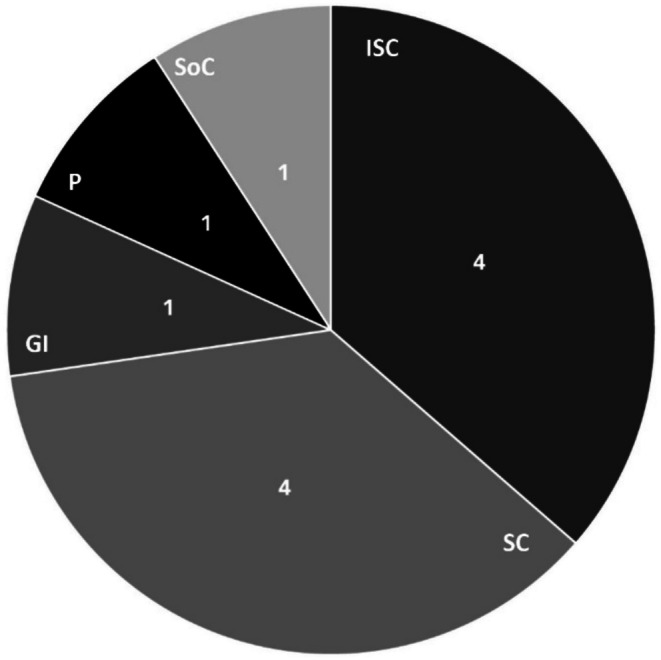
Total adverse events for HCC‐HA_PROF‐B_ by AEs main category, January 1, 2020 to October 31, 2023. AEs are indicated as follows: GI, gastrointestinal conditions (abdominal distension); ISC, injection site conditions (oedema, erythema or swelling); P, pain; SC, skin conditions (erythema, pruritus, rash); SoC, social circumstances (tanning).

The number of AEs per main category for HCC‐HA_PROF‐B_ is presented in Figure 2. A total of 11 AEs were reported between January 1, 2020 and October 31, 2023; the most common types of AEs reported were “administration site conditions” (*n* = 4) and “skin and subcutaneous tissue disorders” (*n* = 4) (Figure 2). The subcategories which contributed to the observed events were administration site conditions (injection site hypersensitivity, *n* = 1; injection site inflammation, *n* = 1; oedema, *n* = 2), skin and subcutaneous tissue disorders (erythema, *n* = 1; idiopathic guttate hypomelanosis, *n* = 1; pruritis, *n* = 1; skin hyperpigmentation, *n* = 1), gastrointestinal disorders (abdominal distension, *n* = 1), musculoskeletal and connective tissue disorders (pain in extremity, *n* = 1) and social circumstances (tanning, *n* = 1). No quality complaints related to the use of HCC‐HA_PROF‐B_ have been reported together with AEs to our knowledge.

Although sale volumes were lower for HCC‐HA_PROF‐B_ due to its recent commercialization, the number of syringes sold increased substantially from the year of commercialization in 2020 to 2023 (Table 3). The number of patients exposed to treatment followed a similar trend to the sales data, with global cumulative patient exposure across the specified time periods estimated at 27692 patients for HCC‐HA_PROF‐B_. The proportion of exposed patients with a safety complaint was low, with 0%–0.013% of patients experiencing a safety complaint across the time periods assessed. The number of patients with a safety complaint generally increased with exposure, but the complaint rate (%) remained low (Table 3).

**TABLE 3:** Patient exposure and complaint rate for HCC‐HA_PROF‐B_ between January 1, 2020 and October 31, 2023.

Year	HCC‐HA_PROF‐B_			
	No. of syringes sold	Estimated no. of patients exposed^a^	No. of safety complaints	Estimated proportion of exposed patients with a safety complaint (%)
2020	100	14	0	0.00
2021	35 922	5132	0	0.00
2022	90 056	12 865	2	0.002
2023	67 766	9681	9	0.013

^a^ The number of patients exposed = number of syringes sold/7.

## 4 Discussion

Although HA injectables are renowned for their favorable safety profiles, AEs remain a pertinent concern. Post‐marketing surveillance, pivotal for monitoring safety and detecting unexpected AEs, has substantiated a low incidence of AE complaints for HCC‐HA_PROF_ and HCC‐HA_PROF‐B_, aligning with previously established safety metrics [20, 21]. However, the reporting of AEs is influenced by the diligence of healthcare professionals, and mild or expected AEs are often underreported. This underreporting is compounded by assumptions in data analyses, such as estimating patient exposure based on the maximum expected usage of syringes, potentially underestimating the actual exposure and, consequently, the incidence rate of AEs. However, the results of this analysis align with the safety profiles of previously published clinical studies for HCC‐HA_PROF_ and HCC‐_HAPROF‐B_ [16]. Similarly, the safety profile established in this analysis aligns with the findings of a systematic review by Kyriazidis et al. 2023, which assessed 48 high‐level randomized controlled studies of HA injectable treatments and reported that most AEs were transient and mild to moderate in severity, and confirmed the positive safety profile of HA fillers [22]. Most reported AEs for HCC‐HA_PROF_ and HCC‐HA_PROF‐B_ were categorized as “general disorders and administration site conditions” and “skin and subcutaneous tissue disorders,” with swelling, redness, and pain being typical and generally transient, occurring within 72 h post‐administration [3, 20]. Serious AEs like vascular occlusion and embolism, though rare, were noted and attributed to incorrect injection techniques rather than the products themselves. This emphasizes the critical need for precise injection techniques and thorough anatomical knowledge to mitigate such risks [23]. Notably, no immune‐mediated hypersensitivity reactions have been directly linked to these products, highlighting their biocompatibility. In high‐risk facial areas, such as the forehead and temples, clinicians must apply advanced knowledge and choose appropriate techniques, such as using cannulas, to reduce the risk of vascular events [24, 25, 26]. These practices are crucial for preventing AEs and improving overall patient outcomes. The steady complaint rate for HCC‐HA_PROF_ despite a significant increase in use, and the low complaint rates for HCC‐HA_PROF‐B_, launched in 2020, suggest a stable safety profile.

While the findings of this study support a favorable safety profile for HCC‐HA_PROF_ and HCC‐HA_PROF‐B_ products, a few limitations regarding postmarketing safety data, in a broader context, should be considered.

First, events that contribute to the safety profile of a product are reported to the manufacturer through the spontaneous initiative of physicians. Reporting both mild and expected AEs, as well as serious or severe AEs, is important for both physicians and patients, as it provides valuable information for the scientific community. However, this process, in particular for mild and expected AEs, is left to a case‐by‐case evaluation, which may lead to underreporting of these events.

Secondly, considering that the majority of the AEs are successfully treated by physician, and in particular those that are a consequence of the product administration, an underreporting to the manufacturer might occur. The lack of reporting may be due to the fact that some of these AEs are described in the product's instructions for use.

Finally, in most cases, patients do not report AEs either because the issue resolves spontaneously or because they have not been adequately trained by the clinician on how to report these issues.

Despite these limitations, when HCC‐HA_PROF_ and HCC‐HA_PROF‐B_ treatments, which are the focus of this study, are administered using appropriate injection techniques and anatomical knowledge, they are well tolerated. While post‐marketing surveillance remains crucial for continuous safety monitoring, the data available thus far suggest that the HCC‐HA_PROF_ and HCC‐HA_PROF‐B_ products demonstrate a stable and robust safety profile.

## 5 Conclusions

Global post‐marketing data show that HCC‐HAPROF and HCC‐HAPROF‐B have favorable safety profiles, with most AEs being predictable and related to administration. While the complaint rate for HCC‐HAPROF has remained stable over time, HCC‐HAPROF‐B has seen a slight increase in complaints, although rates are still low. Continued monitoring is needed to confirm trends as patient exposure increases. Overall, both treatments align with the safety expectations set by earlier clinical studies.

## Conflicts of Interest

G.S., A.T., D.C., and F.A. declare no conflicts of interest in the writing of this manuscript. C.G., F.M., C.C., F.G., and G.B. are currently employees of IBSA Farmaceutici Italia Srl.

## Data Availability

The data that support the findings of this study are available on request from the corresponding author. The data are not publicly available due to privacy or ethical restrictions.
